# Iterative feedback bio-printing-derived cell-laden hydrogel scaffolds with optimal geometrical fidelity and cellular controllability

**DOI:** 10.1038/s41598-018-21274-4

**Published:** 2018-02-12

**Authors:** Ling Wang, Ming-en Xu, Li Luo, Yongyong Zhou, Peijian Si

**Affiliations:** Key Laboratory of Medical Information and 3D Bioprinting of Zhejiang Province, Hangzhou Dianzi University, Hangzhou, 310018 China

## Abstract

For three-dimensional bio-printed cell-laden hydrogel tissue constructs, the well-designed internal porous geometry is tailored to obtain the desired structural and cellular properties. However, significant differences often exist between the designed and as-printed scaffolds because of the inherent characteristics of hydrogels and cells. In this study, an iterative feedback bio-printing (IFBP) approach based on optical coherence tomography (OCT) for the fabrication of cell-laden hydrogel scaffolds with optimal geometrical fidelity and cellular controllability was proposed. A custom-made swept-source OCT (SS-OCT) system was applied to characterize the printed scaffolds quantitatively. Based on the obtained empirical linear formula from the first experimental feedback loop, we defined the most appropriate design constraints and optimized the printing process to improve the geometrical fidelity. The effectiveness of IFBP was verified from the second run using gelatin/alginate hydrogel scaffolds laden with C3A cells. The mismatch of the morphological parameters greatly decreased from 40% to within 7%, which significantly optimized the cell viability, proliferation, and morphology, as well as the representative expression of hepatocyte markers, including *CYP3A4* and albumin, of the printed cell-laden hydrogel scaffolds. The demonstrated protocol paves the way for the mass fabrication of cell-laden hydrogel scaffolds, engineered tissues, and scaled-up applications of the 3D bio-printing technique.

## Introduction

Three-dimensional (3D) bio-printing represents a promising method for fabricating complex 3D tissue or organ constructs through the layer-wise and controllable positioning of cell-containing media^1–3^. Hydrogels have been widely used as cell-laden biomaterials for 3D bio-printing in tissue engineering and regenerative medicine (TERM) applications^4–6^, wherein the designed and printed inner porous architectures play an essential role in the cell growth, tissue formation and function reconstruction of 3D bio-printed tissue scaffolds^7–11^. Therefore, high geometrical fidelity between the designed and as-printed scaffolds enables the efficient utilization of the unique structurally tailored advantages of 3D bio-printing technique to facilitate cellular controllability^12,13^. 3D bio-printing cell-laden hydrogel scaffolds with optimal geometrical fidelity and cellular controllability are of great significance for research on the biofunctional reconstruction of printed cell-laden scaffolds, mass fabrication of engineered tissues and organs, and scaled-up applications of the 3D bio-printing technique.

However, because of the inherent complex variability of hydrogels and cells, the fabrication of 3D bio-printed cell-laden hydrogels with predesigned geometry and desired cellular properties faces many challenges^14–16^, such as unexpected gel deformation^14–18^, uncontrollable cell dynamics^19–22^ and printing process parameters mismatch^23,24^. Recently, researchers have attempted to regulate the printing parameters or hydrogel printability to improve printing accuracy and cytoactivity, e.g., regulating the flow behavior of cell-alginate suspensions^21,22^, modulating shear stress by varying hydrogel viscosity and printing pressures^23^, and optimizing printing parameters based on quantitative 2D image analysis of the printed strands^24^. Most of the above research focused on the relationship between the biomaterials’ physical properties and printing process parameters; however, little attention has been focused on utilizing cybernetics methods to ensure a precise match between the designed and as-printed scaffolds. Furthermore, this research determined printing accuracy using 2D analysis instead of 3D measurements, and the inner printing errors may present different features compared to those on the outer surface. In this study, we developed an iterative feedback bio-printing (IFBP) approach based on the 3D quantification of the printed results to optimize the printing geometrical fidelity and cellular controllability. Moreover, we discussed the influence of printed geometric fidelity on the biological outcome.

The IFBP method is based on accurately and nondestructively detecting the mismatch between the design and as-printed scaffolds, thereby providing quantitative linear feedback control to improve the 3D bio-printing process. Micro-computed tomography (micro-CT) is a commonly used technique to image the inner architecture of engineered tissues and tissue engineering scaffolds^25–28^. However, since the X-ray absorption-based image contrast between culture media and high-moisture hydrogels is very poor, micro-CT imaging is unsuitable for imaging cell-laden hydrogel constructs under standard culture conditions^29^. Recently, optical coherence tomography (OCT) has shown potential for imaging the structure and function of engineered tissues fabricated by cells and bionic extracellular matrices, including natural biopolymer or synthetic polymers such as chitosan, collagen, alginate, Matrigel, polydimethylsiloxane (PDMS), polylactide (PLA), polycaprolactone (PCL) and poly(lactic-co-glycolic acid) (PLGA)^30–33^. Because of its nondestructive detection, high resolution (1–10 μm), deep penetration (1–5 mm) and real-time imaging ability (above 25 frames/s), OCT can be used in the evaluation of the constructs’ structure, cell dynamics and tissue development in engineered tissues^30–38^. Our earlier work established a platform for the automatic quantitative characterization of 3D bio-printed hydrogel scaffolds using swept-source OCT (SS-OCT) imaging, which revealed the linear relationship between the printed and designed geometries^39–41^. Therefore, we present an OCT-IFBP approach that iteratively regulates the mismatches between designed and as-printed scaffolds based on the linear feedback formula obtained experimentally from OCT imaging and analysis.

In this study, eight types of cell-laden hydrogel scaffolds with different pore sizes were designed, printed and quantitatively characterized in the first experimental loop. Two types of tailored cell-laden hydrogel scaffolds were printed with high geometric fidelity and cellular controllability using the OCT-IFBP approach. Gelatin/alginate hydrogels laden with C3A cells were used to investigate the effects of the OCT-IFBP method. An evaluation of the cell-laden hydrogel scaffolds was performed, and comparison between the OCT-IFBP and direct printing methods were based on the cell viability, proliferation, and morphology, as well as the functional expression of hepatocyte markers, such as CYP3A4 and albumin.

## Results

### Printability of a gelatin/alginate-driven hydrogel

**Figure 1:**
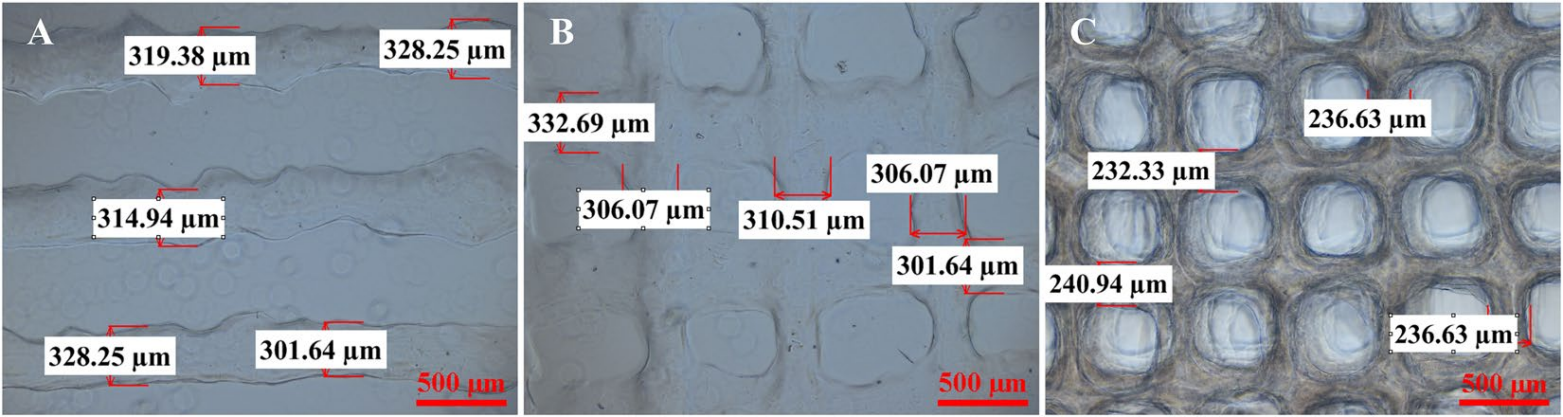
Microscopic images of (**A**) cylindrical filaments (1D), (**B**) lattice sheets (2D) and (**C**) porous scaffold (3D) prepared using the same printing conditions. The strut size and surface roughness of different structures show significant variations.

The printability of the hydrogels significantly affects printing robustness and accuracy. To systematically investigate the printability of hydrogels, different structures, including 1D cylindrical filaments, 2D lattice sheets and 3D grid scaffolds, were printed with the same designed strut size (StS) of 210 μm using the same printing conditions, as described in the Methods section. All printed scaffolds were imaged and characterized using an optical microscope (Ti-U, Nikon, Japan), as shown in Fig. 1. The StSs of the 1D cylindrical filaments, 2D lattice sheets and 3D scaffolds were 318.49 ± 11.04 μm, 311.40 ± 12.31 μm, and 236.63 ± 3.52 μm, respectively. Distinct variations in the mismatch between the designed and as-printed scaffolds were observed. Compared to the 1D and 2D structures, the 3D porous hydrogel scaffolds presented remarkably different deformation properties. Unlike 2D structures, quantifying the mismatches in 3D hydrogel scaffolds is fundamental to optimize printing fidelity.

### Imaging and characterization of 3D bio-printed cell-laden hydrogel scaffolds using OCT

**Figure 2:**
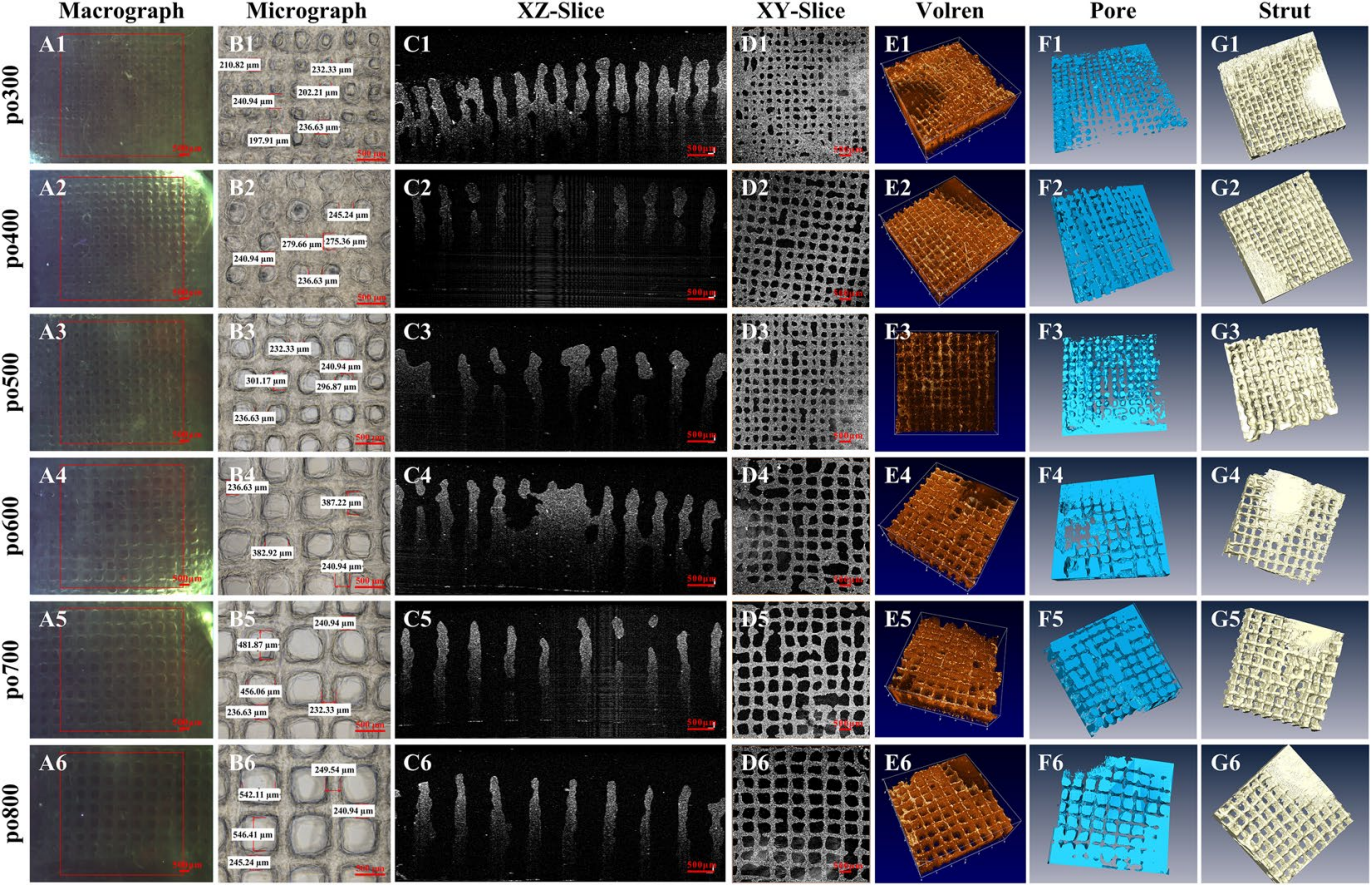
Images of the six different hydrogel cell-laden scaffolds: macrographs (**A1**–**A6**) and micrographs (**B1**–**B6**) showing the surface, OCT cross-sectional images exhibiting an imaging depth of up to 3.0 mm (**C1**–**C6**), OCT en-face images (**D1**–**D6**), and 3D rendering (**E1**–**E6**) and reconstruction (**F1**–**F6**, **G1**–**G6**) showing the variation in the pores and materials.

To investigate the intrinsic characteristics of the mismatch between the designed and as-printed scaffolds, the gelatin/alginate/*C3A* 3D scaffolds were successfully fabricated with different predefined pore sizes (PSs), which evenly increased at regular intervals. All of the 3D scaffolds were imaged and analyzed by our custom-built SS-OCT system. The respective OCT images of six different scaffolds are shown in Fig. 2: A1–A6 show the image range of OCT in the XY plane, while B1–B6 provide the corresponding microscopic images of different hydrogel scaffolds. High-resolution (10 μm) OCT images clearly distinguish the hydrogel area (gray) from the pore area (black) (C1–C6, D1–D6). For the high scattering hydrogel scaffolds, an OCT imaging depth of up to 3.0 mm was achieved (C1–C6, E1–E6), which, in contrast to the 2D microscopy images, is not limited to the outer surface. 3D OCT imaging also enables the reconstruction of pore networks (F1–F6) and material distribution (G1–G6).

Based on our previously proposed algorithm, the PS and StS of the different scaffolds were quantified using the 2D en-face OCT images, whereas volume porosity (VP), surface area (SA) and pore volume (PV) were obtained from the reconstructed 3D images. All the quantitative characterization results were measured based on 5 replicates and are listed in Table 1.

**Table 1:** Quantification structure characterization results of the six different hydrogel scaffolds.

Parameters	po300	po400	po500	po600	po700	po800
PS/μm	206.6 ± 5.4	260.4 ± 6.4	306.6 ± 5.2	372.3 ± 1.7	482.4 ± 5.1	531.3 ± 8.7
StS/μm	235.5 ± 12.2	242.3 ± 9.3	238.4 ± 6.8	240.6 ± 5.1	236.5 ± 14.0	244.8 ± 14.5
VP/%	20.7 ± 1.3	29.0 ± 1.0	35.1 ± 1.7	42.1 ± 2.9	50.2 ± 2.1	51.0 ± 3.2
SA/mm²	527.2 ± 8.6	500.4 ± 7.0	473.5 ± 12.4	400.8 ± 3.7	372.3 ± 12.0	374.4 ± 24.7
PV/mm³	18.6 ± 1.2	26.1 ± 0.9	31.6 ± 1.5	37.9 ± 2.6	45.7 ± 1.4	45.9 ± 2.9

**Figure 3:**
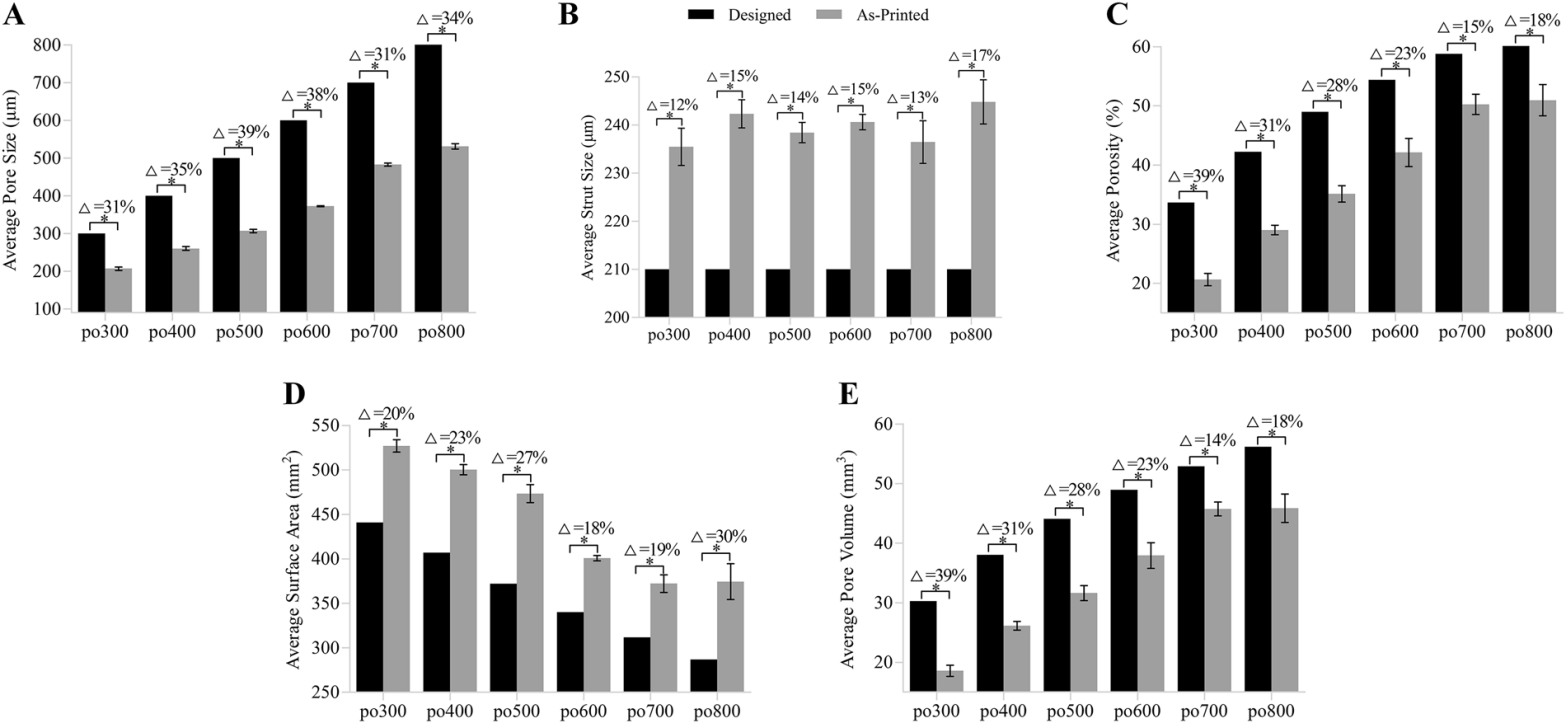
Average PS (**A**), StS (**B**), VP (**C**), Sa (**D**) and PV (**E**) calculated using OCT image analysis compared to the respective design parameters. Error bars represent the measured intra-batch variances. Δ Represents the discrepancy between the design and average as-printed value. Statistical analysis: *p < 0.05.

We compared the morphological parameters of the printed hydrogel scaffolds with their design parameters, as shown in Fig. 3. Data for the arithmetic average of the five sample scaffolds are displayed in the form of a histogram, where the black histogram bars represent the predefined values and the gray histogram bars represent the actual printed values. The percent above the histogram denotes the percentage of the mismatch between the designed and as-printed scaffolds, which is expressed as the absolute value. From the graphs, the difference ranges of PS, StS, VP, SA and PV are 31–39%, 12–17%, 15–39%, 18–30% and 14–39%, respectively, which indicate significant discrepancies between the designed and as-printed scaffolds.

### Determining the feasibility of the OCT-IFBP approach

**Figure 4:**
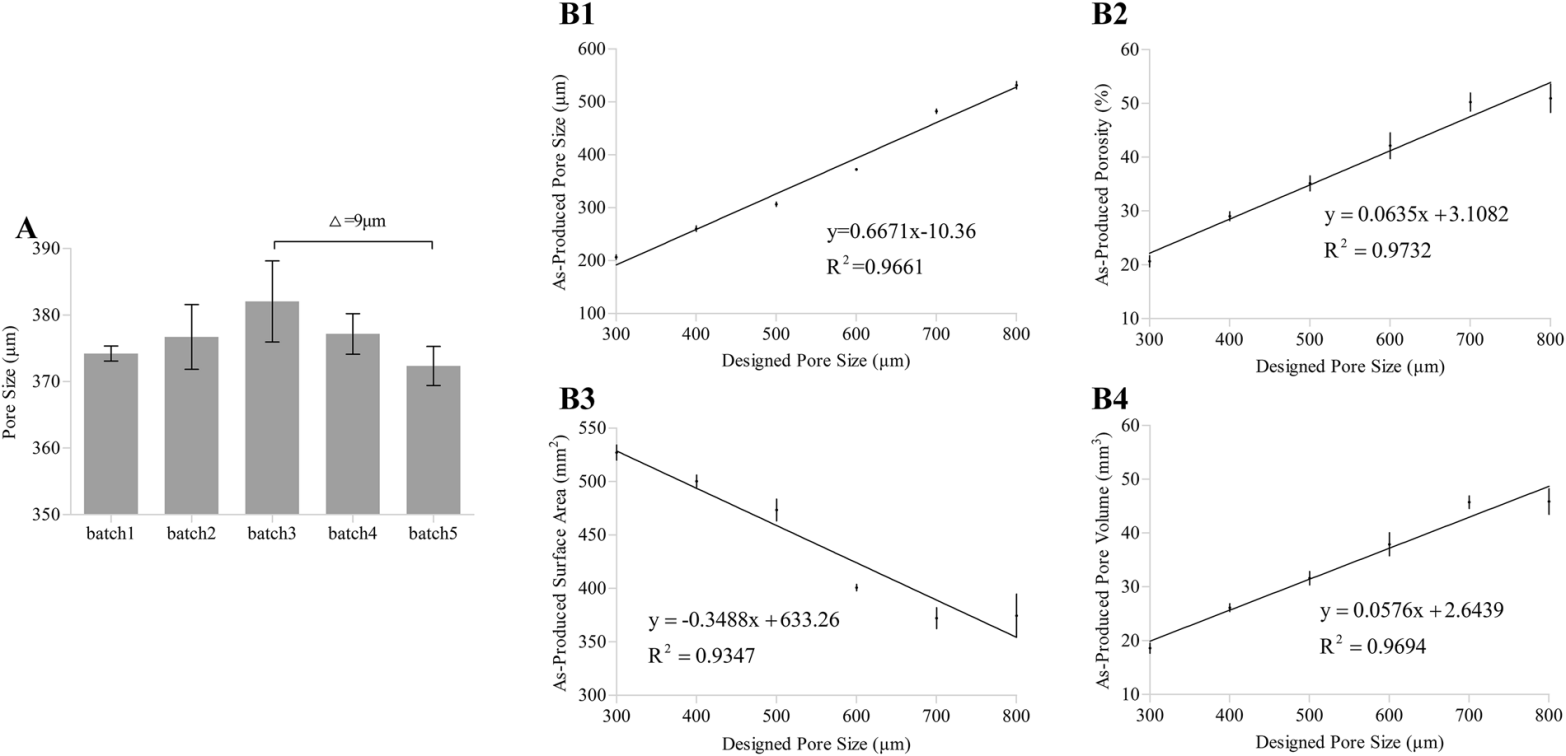
Results of inter-batch variation and intra-batch correlation analysis. (**A**) The average PS for 5 batch experiments of printing po600 spread out over six months and determined using OCT image analysis. Δ is the measured maximal inter-batch variation. (**B1**–**B4**) Correlation between the designed PS and average values of (**B1**) PS, (**B2**) VP, (**B3**) SA, and (**B4**) PV obtained by OCT image analysis (intra-batch variation), which shows the corresponding correlation functions of six different predefined geometries (po300, po400, po500, po600, po700, and po800). Error bars represent the measured intra-batch variation.

To determine the feasibility of the OCT-IFBP approach for the fabrication of cell-laden hydrogel scaffolds with high geometrical fidelity and cellular controllability, it is necessary to investigate both the inter-batch variations and the intra-batch correlations, which determine the robustness and controllability of the 3D bio-printing technique. First, the inter-batch variations are used to determine the repeatability and robustness of our 3D bio-printing technique and process. The repeatability is defined as the variations in the morphological parameters of printed scaffolds with identical designs at different times and in different batches. 3D hydrogel scaffolds with the same designed geometry (po600) were printed at five time points over six months, and the morphological parameters of each batch were quantified by 3D OCT image analysis. As shown in Fig. 4A, the largest significant difference between different batches was only 9 μm of the average PS, which was acceptable from a practical point of view. Therefore, our 3D bio-printing process is robust and repeatable.

The intra-batch correlation analysis was used to determine the fidelity between the designed and as-printed structures. The PS, VP, SA, and PV of six different predefined scaffolds printed in the same batch were analyzed as a function of the preset PS of each. Figure 4B1–B4 show that there is a good linear correlation between the printed values and the designed PS, which is expressed in the linear fitting functions and considered suitable as a feedback to adjust the production run.

### Validation of the effectiveness of OCT-IFBP approach

**Figure 5:**
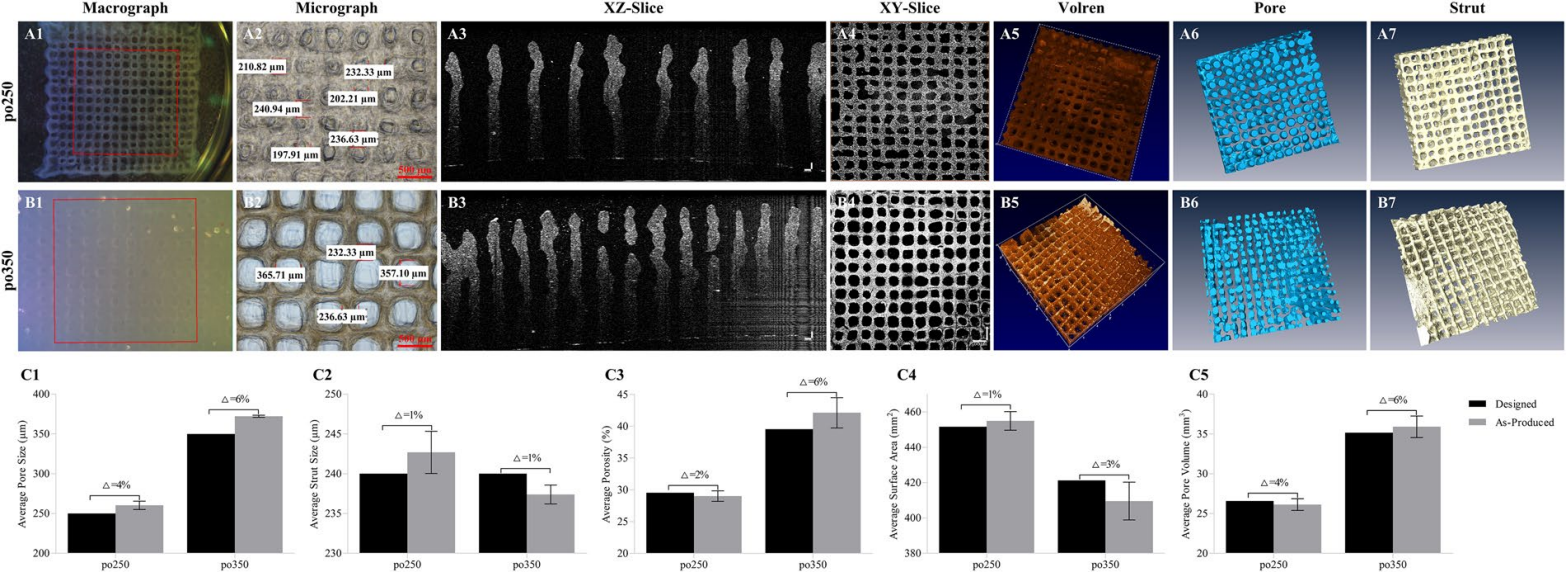
Images and characterization of 3D cell-laden hydrogel scaffolds of po250 and po350 fabricated by OCT IFBP method. (**A1**–**A6**) po250 and (**B1**–**B6**) po350; (**A1**,**B1**) images; (**A2**,**B2**) micrographs showing the surface; (**A3**,**B3**) OCT cross-sectional images with a depth of up to 3.0 mm; (**A4**,**B4**) OCT en-face image; (**A5**,**B5**) 3D rendering of OCT images; (**A6**,**B6**) reconstruction of pore networks; (**A7**,**B7**) reconstruction of material distribution displaying a more uniform pore and strut distribution than the first run; (**C1**) average PS; (**C2**) StS; (**C3**) VP; (**C4**) Sa; (**C5**) and VP obtained from OCT images compared to the corresponding predicted values. Error bars represent the intra-batch variation. Δ Represents the discrepancy between the average as-printed and predictive value.

To verify the ability of OCT-IFBP to print cell-laden hydrogel scaffolds with tailored morphological characteristics, two scaffolds with the envisioned average pore sizes of 250 μm and 350 μm were redesigned and printed with six replicates: po250+ was designed with a PS of 390 μm, and po350+ with a PS of 540 μm. Based on the defects observed in the first experimental run, printing process optimization was also performed to improve printing accuracy. A thermostat was used to ensure the uniformity of the temperature of the dispensing syringe, thus enabling a certain homogeneity in strand distribution. The printing parameters were adjusted based on our previously constructed bioink data to a pressure of 0.43 MPa, an X/Y printing speed of 8.5 mm/s, a nozzle temperature of 8 °C, a platform temperature of 4 °C and a layer height at 100 μm. Figure 5A and B show the microscopy and OCT images of the two scaffolds reprinted according to the feedback from OCT imaging and analysis in the first run.

Figure 5C1–C5 compare the morphological parameters of the reprinted scaffolds measured by 3D OCT analysis with those predicted by the aforementioned empirical correlation functions, represented by the error bars in the intra-batch variation. There are significant differences between the preset parameters and the measured values. However, compared to the printing results in the first run, the mismatches are reduced tremendously, i.e., the PS mismatches decreased from 31–39% to 4–6%; the StS mismatches decreased from 12–17% to 1%; the VP mismatches decreased from 15–39% to 2–6%; the SA mismatches decreased from 18–30% to 1–3%; and the PV mismatches decreased from 14–39% to 4–6%. Therefore, our OCT-IFBP method has significantly improved the geometric fidelity of the 3D bio-printed hydrogel scaffolds.

### *In vitro* cell biological behavior analysis

To verify whether the improvement in geometric fidelity will facilitate cell growth and functional expression, the *in vitro* biological characteristics of two groups of cell-laden hydrogel scaffolds were evaluated and compared based on cell survival and proliferation and relevant functional expression. The improvement groups, defined as po250+ and po350+, used the abovementioned OCT-IFBP method to obtain the desired geometry structures, whereas the controlled groups, which were define as po250 and po350, were printed directly. All the quantitative characterization results of two groups were measured based on 5 replicates and are listed in Table 2.

**Table 2:** Quantification structure characterization of the two different hydrogel constructs (po250 and po350).

Parameters	po250		po350	
	Direct Print	OCT-IFBP	Direct Print	OCT- IFBP
PS/μm	177.1 ± 16.4	260.4 ± 9.1	223.1 ± 6.3	372.3 ± 2.35
StS/μm	237.8 ± 20.5	242.7 ± 4.6	243.5 ± 7.9	237.4 ± 2.0
VP/%	11.0 ± 2.5	29.0 ± 1.4	25.3 ± 1.5	42.1 ± 4.1
SA/mm²	546.1 ± 17.5	454.9 ± 9.0	502.2 ± 5.5	409.5 ± 18.5
PV/mm³	17.0 ± 2.2	26.1 ± 1.3	22.8 ± 1.9	35.9 ± 2.3

### Cell viability and proliferation

**Figure 6:**
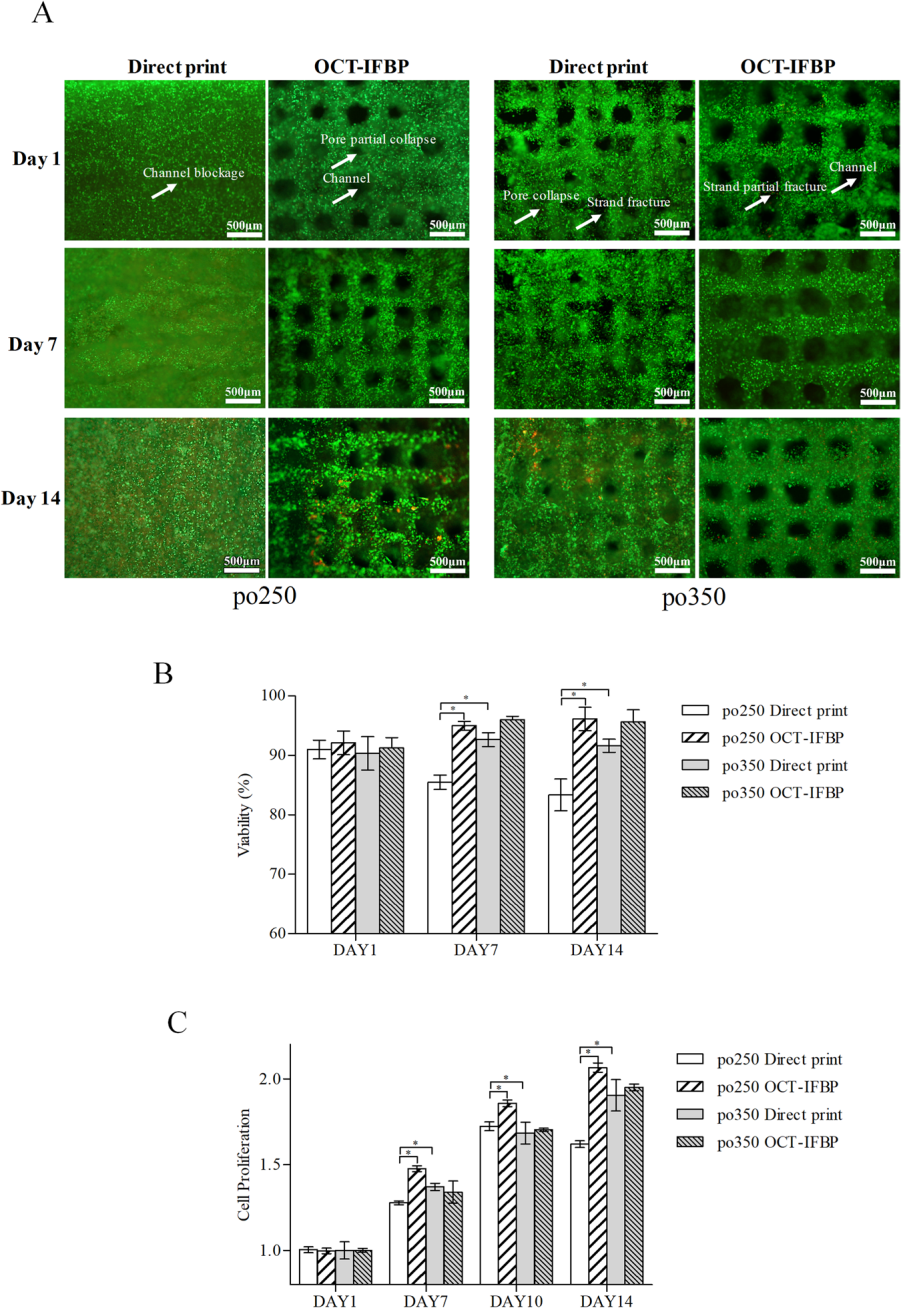
Viability analysis and proliferation assay of 3D cell-laden hydrogel scaffolds fabricated by OCT-IFBP and direct printing method: (**A**) comparison of live/dead staining fluorescence microscopy images between OCT-IFBP and direct printing group (green represents living cells, whereas red represents dead ones); Scale bar = 500 µm (**B**) Comparison of viability; (**C**) comparison of proliferation;. Error bars represent the intra-batch variation.

Figure 6 shows the cell viability and proliferation assay results of the po250 and po350 improvement and controlled groups. The cell viability percentages of po250+ in the improvement group were 92.10 ± 2.78% after 1 day, 94.99 ± 1.03% after 7 days, and 96.13 ± 2.79% after 14 days, whereas the corresponding viability percentages of po250 printed directly were 90.99 ± 2.22%, 85.50 ± 1.70%, and 83.36 ± 3.76% (Fig. 6B). Moreover, the cell viability percentage of po350+ was 90.24 ± 2.45% after 1 day, 96.00 ± 0.83% after 7 days, and 95.64 ± 2.84% after 14 days, whereas the corresponding viability percentages of the po350 in the control group were 90.33 ± 4.00%, 92.64 ± 1.66%, and 91.62 ± 1.57% (Fig. 6B).

To investigate the cell growth characteristics of two groups of 3D bio-printed scaffolds, the alamarBlue assay was performed to analyze cellular metabolic activity on days 1, 7, 10 and 14 (Fig. 6C). The proliferation ratios of po250 were 1.00 ± 0.02 at day 1, 1.28 ± 0.02 at day 7, 1.73 ± 0.04 at day 10, and 1.62 ± 0.03 at day 14, whereas the corresponding proliferation ratios of po250+ were 1.00 ± 0.02, 1.48 ± 0.02, 1.86 ± 0.03 and 2.07 ± 0.05. The proliferation ratios of po350+ in the improvement group were 1.00 ± 0.02 at day 1, 1.34 ± 0.09 at day 7, 1.70 ± 0.02 at day 10, and 1.95 ± 0.03 at day 14, whereas the corresponding proliferation ratios in the po350 control group were 1.00 ± 0.07, 1.37 ± 0.03, 1.68 ± 0.09 and 1.90 ± 0.13. According to the synergistic analysis of viability and proliferation (Fig. 6B,C), an average PS of 250 μm was considered preferable for C3A cell growth within the hydrogel scaffolds.

### Cell distribution and cell morphology

**Figure 7:**
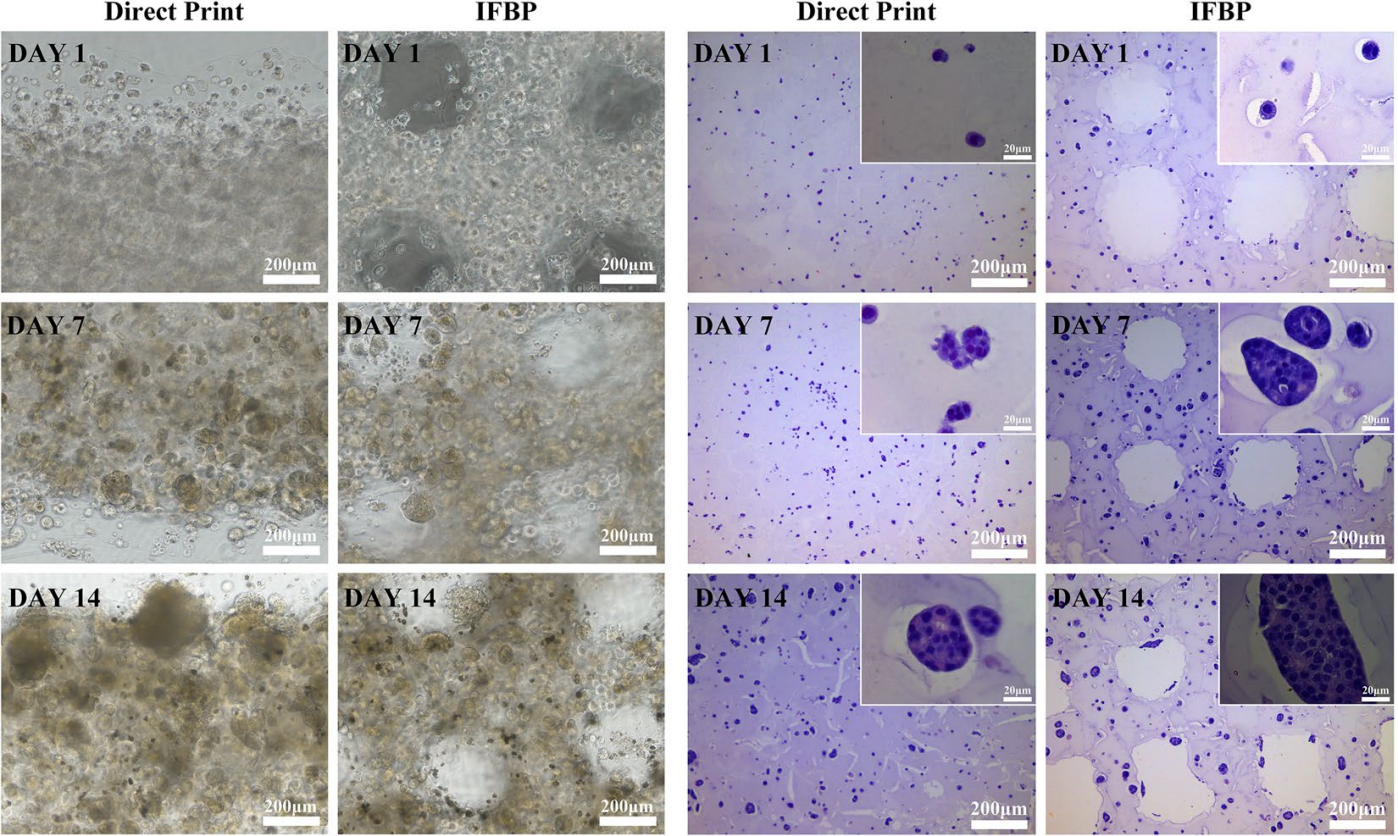
Cell distribution and cell morphology of po250 and po250+: (**A**) DIC microscopy image of cell distribution; (**B**) HE images of cell morphology.

The po250 and po250+ groups were chosen to investigate the improvement effects on other biological properties of the 3D bio-printed cell-laden hydrogel scaffolds. Figure 7 shows the cell distribution obtained with a DIC microscope and cell morphology examined using HE staining of euchromatic nuclei. At all investigated time points, i.e., days 1, 7, and 14, the tailored structures obtained by OCT-IFBP have an evident effect on the cellular spheroid ratios. Furthermore, more cellular spheroids were observed around the pore in the improvement group, whereas the control group exhibited cells that tended to be marginalized and much fewer cellular spheroids.

### Cellular function expression

**Figure 8:**
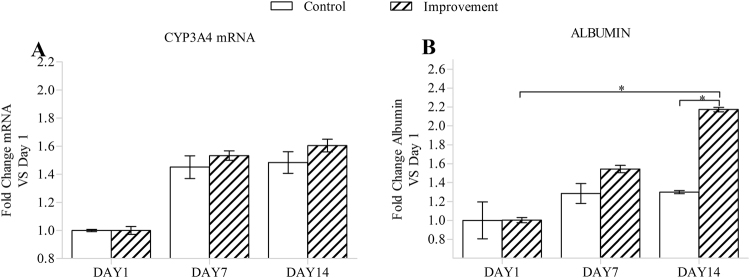
Quantity of the CYP3A4 mRNA (**A**) and albumin (**B**) from hydrogel cell-laden scaffolds. Error bars represent the intra-batch variation. Statistical analysis: *p < 0.05.

The cell’s potential to express liver-specific functions, such as the expression of the *CYP3A4* protein and the production of albumin, was detected and compared between the two groups (Fig. 8). The *albumin* expression in the improvement group increased more sharply over time than that in the control group. The expression of the *CYP3A4* protein in the improvement group was also increased compared to that in the control group. Clearly, the improvement in the geometrical fidelity and controllability enhanced the tissue functionalization of the 3D bio-printed cell-laden hydrogel scaffolds.

## Discussion

For tissue engineering scaffolds fabricated by 3D bio-printing, the internal porous geometries are tailored to preferably mimic the 3D structures of natural tissues and organs. These geometries determine cell growth, functional expression and nurture properties after printing^42–44^. Hydrogels are suitable for 3D bio-printing because they are good biomaterials for cell encapsulation and excellent biomimetics of the human ECM. However, one of the main challenges in the 3D bio-printing of cell-laden hydrogel scaffolds is fabricating porous structures exactly matching predesigned geometries that meet desired biological requirements. Our study aimed to address the problem of how to exploit the unique advantages of the structure customization of the 3D bio-printing technique to control the cell dynamics and functional expression.

Different structures ranging from 1D to 3D were printed to investigate the printability of hydrogels and the characteristics of printing deformation. Our results show different distortions in the 1D, 2D and 3D printed hydrogel scaffolds (Fig. 1). The phenomenon of the deposited strands appearing considerably larger in 2D than in 3D may be partly ascribed to the much greater stiffness of the printing substrate; that is, the 1D and 2D strands were deposited on a glass slice, whereas the 3D strands were deposited on the hydrogels. On the one hand, the printing substrates had different elasticities and exhibited diverse stress effects. On the other hand, the dimensions of the deposited strands may have influenced the deposited surface area and the corresponding swelling degree and cross-linking and further contributed to the accumulation of structural distortion^45^. Other researchers have also demonstrated that 2D sheets and 3D porous scaffolds present different deformation characteristics induced by hydrogel swelling^15^, cross-linking deformation^46^, stress interaction^16^, thermo condensation^17,19,47^, layer height mismatch^14,22^ and other factors^14,46,48^. These findings emphasize the importance of deep and nondestructive imaging for accurately assessing the printing quality of 3D bio-printed cell-laden hydrogels. In this paper, OCT was employed to assess the architecture of 3D bio-printed cell-laden hydrogel scaffolds with a resolution of 6 μm and a depth of 3.0 mm. The ability to image without contact and the low exposure dose of OCT led to almost no impairment of the cells within the hydrogels^29,32–38^. Furthermore, OCT depends on the scattering property of the sample and thus poses low risk to altering the properties and structures of the samples^30–33^. High-resolution OCT images clearly identified the fabrication defects (Fig. 2C1–C6,D1–D6,E1–E6) in the first run and revealed that the inner structure of 3D porous scaffolds exhibited different features compared to the outer surface (Fig. 2C1–C6, E1–E6,F1–F6,G1–G6). Therefore, determining the hydrogel printability or printing accuracy based on 2D analysis instead of 3D measurements is not sufficiently precise for printing process guidance and quality prediction. Furthermore, 3D OCT image analysis enabled accurate quantification of the mismatch between the designed and as-printed scaffolds (Fig. 3). Based on our previously developed automatic quantitative visualization algorithm^39^, both the locations and dimensions of each printing defect could be defined, which was vital to the optimization of the printing process in the second production run.

We successfully controlled the structural parameters of the 3D bio-printed hydrogels to match closely the design parameters (Fig. 5) using the OCT-IFBP method through two closed-loop iterative feedback optimization steps. The first experiment run was used to determine the stability and controllability of the 3D bio-printing system. There was only a 9-μm significant difference in the actual average PS between 6 different batches covering 6 months (Fig. 4A), which demonstrated the repeatability and robustness of our 3D bio-printing system. The design in this run should cover a wide range of geometrical features. PS is an essential variable, as it affects other morphological parameters of the printed scaffolds. Furthermore, PS variations also influence cell growth, morphogenesis, functional expression and nutrient/waste transport properties. Considering these conditions, the preset pore dimensions were varied from 300 μm to 800 μm with evenly distributed intervals. The good linear correlation between the printed morphological parameters and the preset PS (Fig. 4B1–B4) demonstrates the controllability of intra-batch printing, which can be used for predicting the production morphological parameters.

To obtain the desired cell-laden hydrogel scaffolds in the second run, not only the empirical design constraints obtained from the first run but also the appropriate printing process optimization should be used for guiding the new design to avoid systematic fabrication errors. We integrated a detailed printing parameter database regarding the specific bioink^41,49,50^ and the information about the location and dimensions of each printing defect obtained from OCT imaging to fully optimize the printing process configuration by employing, for example, distributed temperature control to ensure homogenous physical gelation of the utilized bioink and precise temperature control of the printing bioink and the printing substrate. Other research has also emphasized the importance of an in-depth parametric study to the printing process^14,21,22,48^.

Importantly, the printing controllability for a PS smaller than 400 μm is poor because of the inherent complex variability of hydrogels and cells. Other researchers have also observed that a scaffold with a PS lower than 600 μm is more vulnerable to pore collapse, even when using a configuration with optimized printing parameters^9,14,46–50^. Figure 2A1–G1 and Fig. 6A in the control group show more pore collapse and strand fracture. Regarding the association between printing robustness and the preset PS, a similar phenomenon was observed in hydrogel scaffolds prepared using either a commercial 3D Bioplotter (Envisiontec, Germany) or another laboratory rapid prototyping system. To obtain a stable structure, designs with a real PS above 500 μm were popular for investigating hydrogel printability and shape fidelity^21–23,43^; however, a PS of 500 μm is too large to translate signals among cells and form tissues^51^. The IFBP approach enables more compact structure in precise printing, for example, the PS in the range of 200~400 μm, which is optimal for nutrient/waste flow, cell migration and angiogenesis^51^.

Many researchers have noticed the influence of the internal geometrical features of a cell-laden scaffold on the biological outcome^51^. As illustrated in Fig. 6, our findings stress the importance of precisely tailored geometric structure for obtaining the desired biological outcomes. Under the optimal printing parameter configuration, the initial cell viability of po250 and po350 did not show a significant difference between OCT-IFBP and direct printing. However, over time, the cell survival in the improvement group seemed to increase compared to that in the control group (Fig. 6A,B,C), with po250 presenting a more obvious improvement in cell growth activity than po350. Comparing the tailored PS 250 μm and 350 μm cell-laden hydrogel scaffolds fabricated by IFBP-OCT (Fig. 6B,C) revealed that the former scaffold showed better cell viability and proliferation. In other words, improving the geometric fidelity of printing is beneficial to feedback optimization of scaffold design and study the fundamental relationship of geometrical structure and cell growth. Furthermore, HE images of the cell-laden scaffolds show a more uniform cell distribution and higher cellular spheroid ratio in the improved po250+ matrix materials than in po250 (Fig. 7). A round cell morphology is typical and preferential for encapsulated C3A cells, and homogeneous cell distribution facilitates tissue formation. The scaffolds provide the cells more favorable microenvironment, which is a better choice for spheroid formation. Moreover, the spheroid has well established cell-to-cell contacts that can preserve cell viability and promote cell functional expression. However, the overall size of the spheroid is smaller in po250 than in the improved po250+, because of the limit of the diffusion of oxygen, nutrients and metabolic waste products, which cause by the different pore sizes.

Although cell viability, proliferation, and morphogenesis are important criteria for characterizing cell growth in engineered tissue, they do not allow an overall evaluation of the improved effect on the biological outcome of a cell-laden hydrogel scaffold. To validate the quality of the cell-laden hydrogel scaffolds fabricated by OCT-IFBP, demonstrating the cells’ capability of displaying normal cell behavior post-construction of the scaffold was necessary. For C3A cells, a hepatocellular carcinoma cell line, this behavior includes maintained expression of hepatocyte markers. The improvement group prepared using the OCT-IFBP method and the control group printed directly were therefore analyzed and compared based on the expression of CYP3A4 and albumin. More active cell function expression was confirmed in the OCT-IFBP group, whereas the expression of CYP3A4 and albumin in po250+ increased more sharply than those in po250 over time (Fig. 8). Therefore, the improvement in the geometrical fidelity and controllability did enhance tissue functionalization of the 3D bio-printed cell-laden hydrogel scaffolds.

OCT-IFBP is furthermore applicable to other 3D bio-printing techniques. On condition that inter-batch and intra-batch variability of the chosen bio-printing technique or systems remains low, other 3D bio-printing technique or systems like inkjet bioprinter, microextrusion bioprinter, laser-assisted bioprinter, whether laboratory or commercial bioprinting machines could replace our used bioprinter in this protocol. OCT is a non-invasive real-time biomedical imaging modality which has been widely used in clinical testing and biomaterial researches. Further, OCT is easy-operating and commercially available, such as SSOCT-1310 (OCTMI Corporation, Irvine, USA), IVS-2000-LC (Santec Corporation, Komaki, Japan), SS-1310 (Moptim Corporation, Shenzhen, China) and so on. The research labs related to 3D bio-printing technique are able to integrate OCT into bioprinting machine quite conveniently and establish an IFBP system based on a home-developing or commercialized OCT machine.

Although the OCT-IFBP protocol is conducive to fabricating a porous cell-laden hydrogel construct with tailored-characteristics, there are still several limitations to this study. The structures that we designed were homogeneous, and the quantitative analysis was based on whole samples. The automatic 3D labeling quantitative characterization algorithm demonstrated in our previous work^39^ can be used to assess a local unit cell and precisely improve the printing geometric fidelity of a heterogeneous design, which will be the subject of our next research direction.

## Conclusions

This study presented an OCT-IFBP protocol for the 3D bio-printing of cell-laden hydrogel scaffolds with high geometric fidelity and cellular controllability. 3D OCT image analysis illustrated that the directly printed cell-laden hydrogel scaffolds had low geometrical fidelity. Inter-batch quantitative characterization and intra-batch linear correlation analysis confirmed good the repeatability and controllability of our 3D bio-printing system and technique, which corroborated the feasibility of OCT-IFBP. Based on OCT imaging and analysis, the quantitative linear feedback design and the printing parameter optimization were integrated to avoid systematic fabrication errors, and the desired printing results were obtained in the second run. The more active cell growth and functional expression when using OCT-IFBP instead of direct printing confirmed that improving the geometric fidelity facilitates the optimization of the cellular controllability of 3D bio-printed cell-laden hydrogel scaffolds through the use of tailored structures.

The novelty of this protocol lies in using OCT, a 3D quantitative characterization method, for iterative feedback design and printing control, thereby optimizing the geometric fidelity and cellular controllability of the 3D bio-printing technique. The demonstrated OCT-IFBP protocol may pave the way for the mass fabrication of cell-laden hydrogel scaffolds, engineered tissues, and scaled-up applications of the 3D bio-printing technique. OCT-IFBP also facilitate optimizing design to obtain desired properties of printed constructs, and investigating the quantitative relationship between scaffold structure and function.

## Methods

### Overview of the OCT-IFBP approach for 3D bio-printing

**Figure 9:**
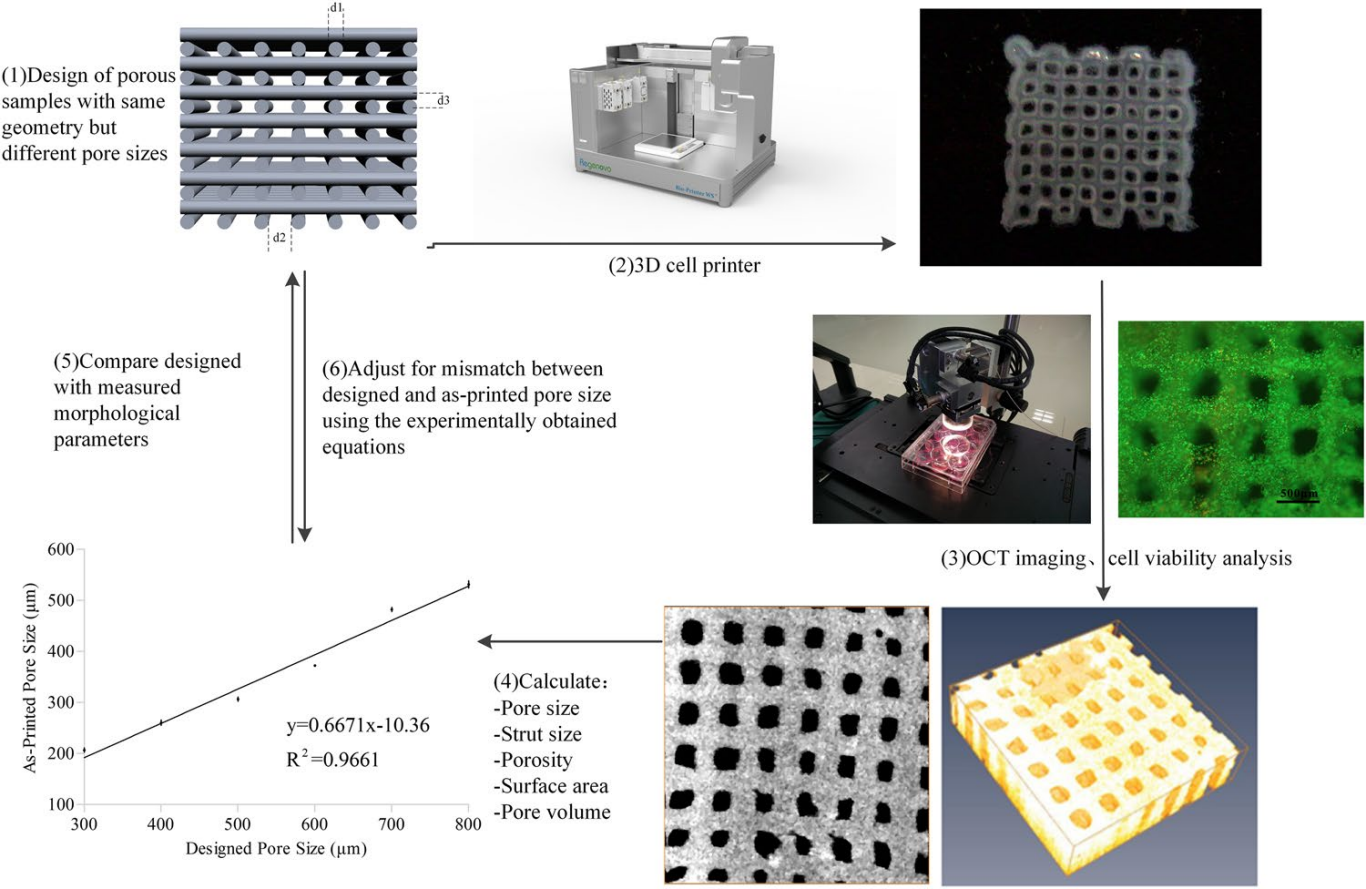
Schematic overview of an OCT-IFBP approach for the 3D bio-printing of cell-laden hydrogel scaffolds with high geometric fidelity and cellular controllability.

An OCT-IFBP approach is applied to optimize the geometric fidelity and cellular controllability of 3D bio-printing for TERM applications. The feedback loop consists of the design, fabrication, overall morphological quantitative characterization and cell behavior analysis of 3D bio-printed cell-laden hydrogel scaffolds. The feedback loop control was performed twice (Fig. 9). The first run was used to evaluate the intrinsic controllability of the 3D printing system and process. In the first run, cell-laden hydrogel scaffolds with different pore sizes were designed and fabricated by the 3D bio-printing system and later imaged and analyzed by a custom-built SS-OCT system. The morphological parameters, including PS, StS, VP, SA, PV, were quantified and compared with the predesigned parameters. The quantitative mismatches between the designed and as-printed scaffolds were used to guide the second run of printing, in which analysis of the correlation between the printed parameters and preset variables and 3D printing process optimization were integrated to decrease the extent of the mismatch. The cellular controllability of the printed scaffolds was investigated by comparing the cellular characteristics of scaffolds printed using OCT-IFBP with those printed directly.

Table 3 shows ten types of designed geometric structures for different evaluation experiments, including OCT image analysis, repeatability studies, specific design constraints, cell viability assays, cell proliferation assays and other biological characterization techniques. The designed geometrical structures were defined according to the PS as po250, po250+, po300, po350, po350+, po400, po500, po600, po700, and po800. In the OCT image analysis experiments of the first run, five printed samples were randomly selected from one of the six types of scaffolds for testing. In the repeatability study, the hydrogel scaffolds of po600 were fabricated 5 different times and were compared quantitatively with the significant differences in the morphological parameters. The specific design constraint experiments were used to verify whether the OCT-IFBP method could significantly reduce the mismatch between the designed and as-printed scaffolds. The related cell viability, proliferation, and biological characterization experiments were used to determine whether optimizing the internal structure fidelity would improve the cellular controllability and whether cellular controllability would contribute to the biofunctional reconstruction of the printed cell-laden hydrogel engineered tissue.

**Table 3:** Overview of the 10 design scaffolds.

Design	po250	po250+	po300	po350	po350+	po400	po500	po600	po700	po800
Pore size(d2)/μm	250	390	300	350	540	400	500	600	700	800
Strut size(d1)/μm	210	235	210	210	235	210	210	210	210	210
Layer thickness(d3)/μm	210	220	210	210	220	210	210	210	210	210
OCT image analysis(n = 5)			√			√	√	√	√	√
Repeatability study (n = 5) (#time = 5)								√		
Specific design constraint (n = 5)		√			√					
Cell viability assay (n = 3)	√	√		√	√					
Cell proliferation assay (n = 5)	√	√		√	√					
Cell morphology assay (n = 3)	√	√								
*CYP3A4/ALB* assay (n = 3)	√	√								

√ indicates that the design was used in the corresponding study.

### Cell culture

C3A liver cells (ATCC^®^ -10741^TM^, American Type Culture Collection, USA), were cultured and maintained in a humidified atmosphere of 95% air/5% CO_2_ at 37 °C with culture medium, which consisted of DMEM, fetal bovine serum (10%, FBS) and penicillin/streptomycin (1%, P/S). All of the agents were obtained from ATCC. The cultivation medium was refreshed every 2 days.

### Design of hydrogel scaffolds

According to previous work, scaffolds with orthogonal interconnected square pores are effective in cell encapsulation. For the purpose of this study, ten types of square pores of different sizes were designed by SolidWorks software. As shown in Fig. 9, d2 represents the PS of the scaffold, and po300 denotes that the design value of PS is 300 μm; d1 represents the StS of the scaffold; and d3 represents the thickness of the layer. The values of d1 and d3 are set at 210 μm. The shape of the scaffold is a cuboid with dimensions of 10 × 10 × 3 mm.

### 3D bio-printing of cell-laden hydrogel scaffolds

The 3D scaffolds were fabricated by sequential fiber deposition using our 3D bio-printing system (Bio-Architect®-Pro, Regenovo Corporation, Hangzhou, China), as described previously^5,33^. Briefly, the hydrogel was prepared with 10% gelatin and 2% sodium alginate mixed at a volume ratio of 1:1. Gelatin and sodium alginate were obtained from Sigma and prepared according to polymer weight/solvent volume (w/v). Next, the prepared hydrogels were mixed with C3A cells to obtain a cell encapsulating density of 1 × 10^6^ cells mL^−1^. The cell-gel mixtures were transferred into a sterilized syringe, loaded into the Bio-Architect®-Pro and cooled to 8 °C. The cell-hydrogel suspensions were extruded from a refit pneumatic nozzle at a pressure of 0.42 MPa and an X/Y speed of 8 mm/s. All of the printing parameters were optimized based on our earlier hydrogel printability experiments^49,50,52–54^. Upon the completion of printing, the scaffolds were cross-linked with 2% CaCl_2_ solution, transferred to 24-well culture plates, washed with DMEM three times and maintained in 1.6 mL of cultivation medium in a CO_2_ incubator at 37 °C. The medium was refreshed daily. The fabricated scaffolds were examined using a light microscope (Ti-U, Nikon, Japan).

PSs of 250 μm and 350 μm were chosen as the typical geometry design to examine whether the OCT-IFBP method benefits the cell growth and functional expression in 3D bio-printed cell-laden hydrogel scaffolds. In the control group, po250 and po350 were printed as described above. For the improvement group, OCT-IFBP was performed with the integration of the design constraint and printing process parametric optimization obtained from the first feedback loop. Thus, the 3D cell-laden hydrogel scaffolds with a desired PS of 250 μm and 350 μm were obtained, which were defined as po250+ and po350+, respectively.

### Imaging and characterization of hydrogel scaffolds using SS-OCT

The 3D bio-printed hydrogel scaffolds were imaged by a custom-built SS-OCT system, which used a swept-source laser with a scanning rate of 50 kHz and a spectrum range of 1310 ± 60 nm. More details on the system can be found in a previous publication^39,55–57^. The 3D OCT images of 6 × 6 × 3.5 mm (1024 × 1024 × 512 pixels) were obtained without contacting the hydrogel scaffolds, which were immersion-cultured in a sealed 24-well culture plate. The covers of the culture plate were custom designed to improve the image quality. The morphological parameters, including PS, StS, VP, Sa, and PV, were quantified based on our previously developed automated characterizing algorithm^39–41^.

### Live/dead staining for testing cell viability

To visualize and analyze the cell viability on the printed scaffolds, live/dead fluorescent staining was performed 1, 7, and 14 days after printing. The printed C3A/hydrogel scaffolds were stained with a mixture of Calcein-AM (Dojindo; 1 μmol/ml) and PI (Sigma; 2 μmol/ml) at 37 °C in the dark for 30 min, gently washed with PBS to remove excess dye, and visualized under an optical microscope (Ti-U, Nikon, Japan). Five images from different areas of three bio-printed scaffolds were chosen randomly. The number of live and dead cells was counted using ImageJ (National Institutes of Health, USA), and the cell viability was calculated as

$${Cell}\,{viability}=\frac{{number}\,{of}\,{green}\,{stained}\,{cells}}{{number}\,{of}\,{green}\,{stained}\,{cells}+{number}\,{of}\,{red}\,{stained}\,{cells}}\times 100 \% $$

### Cell proliferation analysis

Cell proliferation was analyzed using the alamarBlue assay, which introduces a fluorometric growth indicator to evaluate cellular metabolic activity. The cell-laden scaffolds were incubated in 1,000 μL of 10% alamarBlue for 2 hours on days 1, 7 and 14 of the study. The fluorescence was detected using a 520-nm excitation wavelength and a 590-nm emission wavelength. The cell number was obtained using a calibration curve determined by correlating a known cell number with the fluorescent intensity of the solution. Five independent samples were tested in each group.

### Representation of the cell morphology in cell-laden scaffolds

Three cell-laden hydrogel scaffolds were treated with a fixative agent (4% (w/v) paraformaldehyde neutral/phosphate (E672002, Sangon Biotech) buffer solution) for 2 hours at room temperature. After 2 hours of fixation, the scaffolds were dehydrated with 70% ethanol and subsequently paraffin-embedded. These specimens were cut into 5-μm-thick slices using a microtome (Leitz, Western German), stained with hematoxylin-eosin (HE) and observed under an optical microscope (Ti-U, Nikon, Japan).

### RNA isolation and mRNA quantitation

Total RNA extraction from the cell-laden scaffolds was performed with Trizol (Invitrogen) according to the manufacturer’s instructions. The quality and quantity of RNA were evaluated with a spectrophotometer (ND-3000, NanoDrop Technologies, DE). The primers of *CYP3A4* and albumin were designed as listed in Table 4 using Primer 5.0 software (Premier Biosoft International, Canada). RT-RNA amplification reactions used 1 µg of total RNA via M-MLV Reverse Transcriptase (Takara Bio. Inc., Japan) to obtain 20 µL of product, from which 2 µL was extracted for qRT-PCR. The qRT-PCR assay was performed on an RT-PCR system (ABI 7300Plus^TM^, PerkinElmer Applied Biosystems, USA) with a special PCR kit (SYBR^®^ *Premix* Ex Taq^TM^ II, Takara Bio. Inc., Japan). The qRT-PCR assay steps consisted of denaturation for 30 s @ 95 °C, 40 cycles of amplification for 5 s @ 95 °C and 34 s @ 60 °C, and melting temperature-determining dissociation for 15 s @ 95 °C, 1 min @ 60 °C, and 15 s @ 95 °C. The assay results were calculated using the ΔΔCT method, and the expression values were normalized to *GAPDH*. All qRT-PCR assays were repeated five times.

**Table 4:** Primers used for real-time PCR analysis.

Gene name	Primer name	Sequence (5′-3′)	Length (nt)	Product size (bp)
*GAPDH*	FP	GGAGCGAGATCCCTCCAAAAT	21	197
	RP	GGCTGTTGTCATACTTCTCATGG	23	
*CYP3A4*	FP	AAGTCGCCTCGAAGATACACA	21	214
	RP	CTGCTGGACATCAGGGTGAG	20	
*ALB*	FP	CGCTATTAGTTCGTTACAC	19	197
	RP	GTCACTTACTGGCGTTTT	18	

FP: forward primer; R: reverse primer.

### Statistical analysis

Unless stated otherwise, all characterization was performed at least in triplicate. The quantitative analysis was based on the measurement of multiple samples (n = 5), and the result of the measurement was analyzed by variance analysis. Tests of statistical significance were performed with SPSS Statistics. Quantitative data is expressed as mean ± SD and re-analyzed by two-way ANOVA. Multiple comparison between the groups was performed using S-N-K method. Statistical significance was set at a level of P < 0.05.
